# The spread in time and space of COVID-19 pandemic waves: the Italian experience from mortality data analyses

**DOI:** 10.3389/fpubh.2024.1324033

**Published:** 2024-02-28

**Authors:** Daniele del Re, Luigi Palla, Paolo Meridiani, Livia Soffi, Michele Tancredi Loiudice, Martina Antinozzi, Maria Sofia Cattaruzza

**Affiliations:** ^1^Department of Physics, Sapienza University, Rome, Italy; ^2^Department of Public Health and Infectious Diseases, Sapienza University, Rome, Italy; ^3^Istituto Nazionale Fisica Nucleare, Sezione di Roma, Rome, Italy; ^4^Department of Developmental and Social Psychology, Faculty of Medicine and Psychology, Sapienza University of Rome, Rome, Italy

**Keywords:** COVID-19, mortality, containment measures, Italy, pandemic, EuroMOMO

## Abstract

**Introduction:**

Italy was the first European country affected by COVID-19. Thanks to governmental containment measures (9 March 2020), the spread of COVID-19 was limited. However, in this context, accurate data assessment is crucial and mortality is a more reliable indicator of the virus spread compared to the count of positive cases. This study aimed to retrospectively evaluate the impact of the pandemic in different areas of Italy using the time series analysis of official deaths and excess COVID-19 deaths.

**Methods:**

Mortality data (23 February–30 April 2022) by Istituto Nazionale di Statistica (ISTAT) were analyzed, including four waves of COVID-19. Previous mortality data (January 2015–November 2019) were used to estimate a Poisson regression model of the pre-pandemic mortality pattern and derive the excess COVID-19 deaths as the difference between the actual deaths number and the extrapolation of the previous mortality pattern to the pandemic period, separately for Northern, Central, and Southern Italy, to compare the impact of mortality across time periods and geographical areas.

**Results:**

Estimated excess compared with official COVID-19 mortality shows that, during the first wave, there was an underestimation of deaths. COVID-19 mortality rate almost doubled the official rate in the North (1.60‰ vs. 0.86‰) and nearly tripled it in the South (0.22‰ vs. 0.08‰). In late 2020-early 2021, official and estimated mortality curves are closer, displaying just a small gap at the start of the second wave. During the fourth wave (end of 2021-early 2022), Northern and Central Italy show reasonable agreement; the South presents a large relative underestimation of deaths (+90% increase), with a large increase in its excess deaths national quota, 9% in the first wave to 42% in the fourth.

**Discussion:**

The results provide a measure of the COVID-19 excess deaths and an unbiased estimate of Italian mortality rates. In the first wave, the gap between official COVID-19 and excess mortality was particularly high and lockdown measures may have reduced the spread of the infection. In the fourth wave, the gap for the South increases again, probably because the healthcare system may not have coped with the prolonged pressure of the pandemic, or for a decreased compliance with the official paper-based mortality surveillance system that could be overcome in the future by digitalizing the process.

## 1 Introduction

Coronavirus disease 2019 (COVID-19), started in China, rapidly became a global emergency, and was declared a pandemic by the World Health Organization (WHO) on the 11th of March 2020 (1). The pandemic was then declared to be ceased on the 5th of May 2023 (2).

Over 770 million confirmed cases, and nearly 7 million deaths have been recorded in the world.

Italy was the first European country to be affected with very high rates of contagion and death, especially in the North of the country. In continental Europe, Italy ranked third with about 26 million as number of cumulative cases, after France and Germany with about 38 million each. According to WHO’s Coronavirus Dashboard, as of 5 May 2023, after the official declaration of the pandemic ending, considering mortality, Italy ranked first with more than 190.000 cumulative deaths followed by Germany with more than 174.000 and France with approximately 168.000 (3).

At the start of the pandemic, due to the rapid spread of the disease overwhelming the healthcare system, the Italian government imposed a nationwide lockdown on the 9th of March 2020 which ended on the 3rd of May 2020 (4). Thanks to the containing measures implemented by the government, the disease was thus limited, and the healthcare system was mostly able to deal with the emergency, although approximately 250,000 confirmed cases and 35,000 deaths were reported in the first wave, which occurred mainly in the north of Italy (5).

The accurate assessment of data is crucial for monitoring the spread of any viruses and informing the public and the policymakers and to direct actions toward individual and collective decisions aimed to control the pandemic (6). During the COVID-19 pandemic, there was much debate about surveillance systems and monitoring data. Different types of data (count of reported COVID-19-positive cases, number of infected and hospitalized patients, patients admitted to intensive care units (ICUs), and number of deaths) have been used, depending on the specific objective being pursued, bearing in mind that there are advantages, and disadvantages to any surveillance system that need to be taken into account when interpreting the data: the number of new positive cases recorded per day heavily depends on the number of tests administered to the population which varies with the spread of the disease (7); the relative number of unreported cases (e.g., cases identified by the individual but not reported to public health authority) increased with the increasing COVID-19 contagiousness but also with the reduction of symptoms related to the diffusion of vaccinations making the number of official daily or weekly cases even less reliable. Hence, mortality data may represent an alternative and reliable source also for COVID-19 as it is for seasonal influenza surveillance (8–10). It is however of extreme importance to precisely identify and detect the specific COVID-19 mortality contribution.

All these considered, monitoring the mortality trends of COVID-19 in the population is still important today to control for the possible onset of new virus variants, which can act as an alert for public health policymakers.

Previous study on excess mortality has been conducted both on Italian data (11–15) and data from other countries (16) and comparing mortality across countries (17, 18), mostly focusing on the first COVID-19 waves in 2020 (12–15, 18, 19) and looking at socio-demographic determinants/correlates of mortality (11).

In this context, our study aimed to investigate COVID-19 Italian mortality data, estimating the excess mortality due to COVID-19 and comparing it with the official mortality during all four Italian waves, up until 30 April 2022. An additional aim was to assess the relationship between COVID-19 mortality and national lockdown/restrictions imposed during the first, second, and third waves of the epidemic in different Italian geographic areas, namely, Northern, Central, and Southern Italy, seeking any novel insight through these data on the general performance of the Italian public health system across time and geographic areas of the pandemic.

## 2 Materials and methods

The first (23 February 2020–30 April 2020), the second (6 October 2020–5 January 2021), the third (1 March 2021–23 May 2021), and the fourth (1 November 2021–30 April 2022) COVID-19 waves have been studied separately with the same analysis procedure. We used data provided by the Italian National Statistical Institute (ISTAT Istituto Nazionale di Statistica) publicly available on their site. Mortality statistics are submitted to the Civil Status Offices of the municipalities. Data refer to the period 1 February 2020–31 July 2022, and the analysis has been performed using data from 23rd February (start of the first wave) up to 30 April 2022 (end of the fourth wave) (20). After the statistical analysis, the results are compared with the official laboratory-confirmed COVID-19 deaths provided by Istituto Superiore di Sanità (ISS) (21). Mortality data by ISS were collected during the pandemic in addition to the ISTAT death forms flow. Indeed, considering the exceptional nature of the pandemic and the state of emergency that has arisen, the Italian Ministry of Health issued the note 0007922 (22), commissioning the ISS to build a collection and review system on a sample of medical records transmitted to the Institute by the Regions to evaluate the main characteristics of COVID-19 deaths. The software used to perform the statistical analysis is *ROOT* (23).

From ISTAT data, the mortality due to COVID-19 was identified and enucleated from all other causes as in a counting process with the identification and discrimination of “signal” and “background” components. The COVID-19 deaths represent the signal, while any other source of mortality represents the background. The background contribution is subtracted from the total observed mortality using statistical methods, based on a maximum-likelihood fit, and it is called in the following as the “baseline.” To determine the baseline an approach similar to the one employed for the analysis of European mortality by EuroMOMO (24) is performed. This is summarized by the following regression model for counts:


$$\begin{array}{l} b\left[i\right]=\mathrm{offset}*\left(1+\mathrm{slope}*i\right)+\mathrm{amplitude}\\ \;*\cos\left(\left(i+\mathrm{phase}\right)*2\pi /365\right) \end{array}$$

where *b*[*i*] is the estimated number of deaths for day i from 01/01/2015 to 30/11/2019; the seasonal component is parameterized by a cosine curve with a period of 1 year, and the amplitude, the phase, and the offset are estimated from data. The offset is multiplied by a linear function to account for possible year-dependent mortality variations due to changes in the age structure of the population. The parameters of this model have been estimated from pre-COVID data corresponding to periods which do not overlap with influenza and heat waves, that is, April–May and September–November of 2015, 2016, 2017, 2018, and 2019 years.

This baseline fitted contribution has been then extrapolated to the COVID-19 wave periods and subtracted from the data to obtain the estimate of the COVID-19 excess of deaths.


$$\mathrm{excess}\left[i\right]=n\left[i\right]-b\left[i\right]$$

where *n*[*i*] is the number of deaths for day i. The fit of the baseline has been made for Italy as a whole and for the Northern, Central, and Southern Italy breakdowns.

Mortality rates (per 1,000) are calculated as the number of deaths divided by the resident population. The uncertainties of the death excess are evaluated taking into account the Poissonian error on n [i] (i.e., $\sqrt{n\left[i\right]}$ ) and the statistical uncertainty from the fit of baseline estimation *b*[*i*].

This methodology is applied to data with different breakdowns: nationwide, Northern, Central, Southern Italy, regional, and provincial levels. These estimated numbers are then compared with the official laboratory-confirmed COVID-19 deaths (21).

## 3 Results

**Figure 1:**
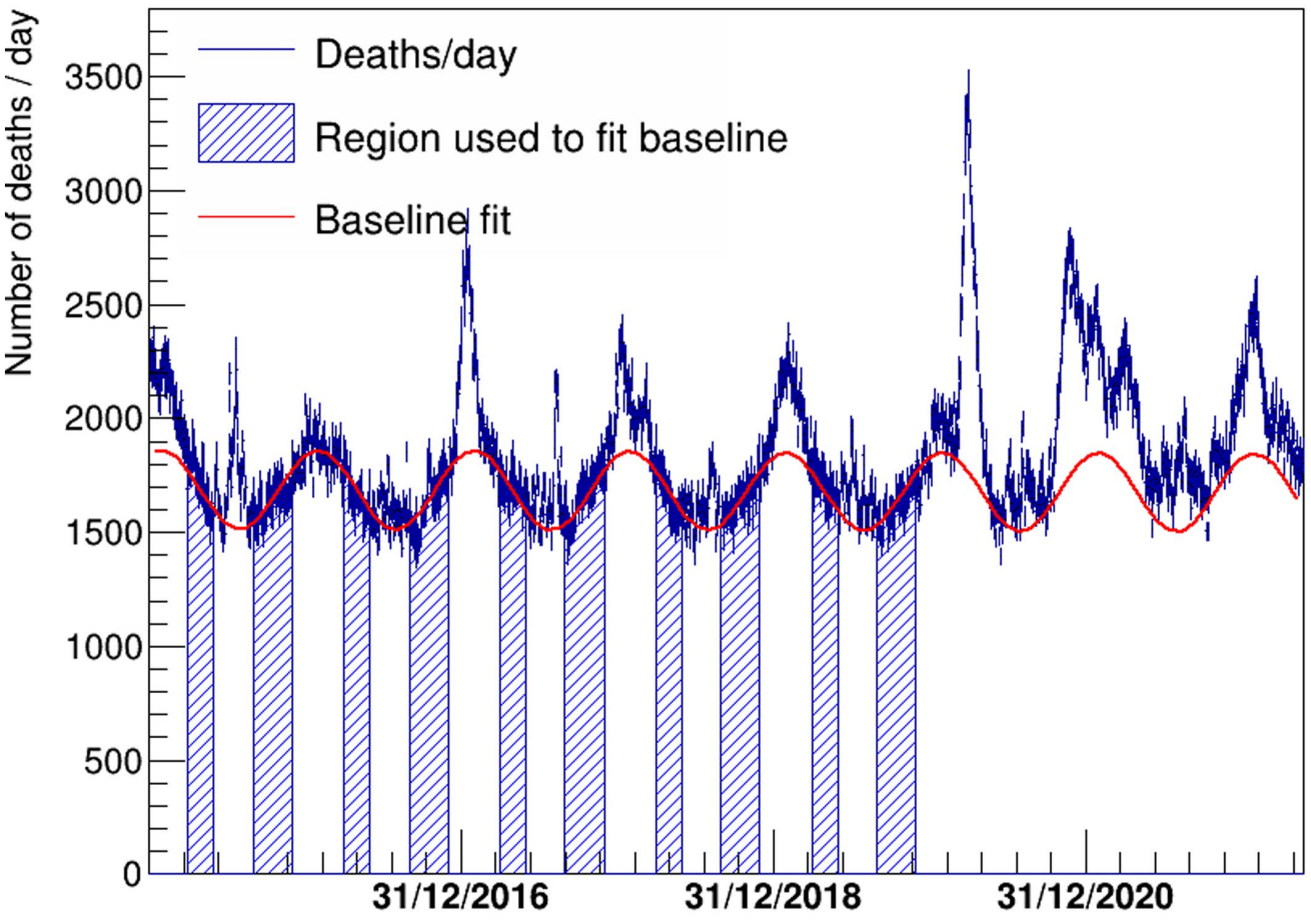
Deaths per day in Italy since 1 January 2015 shown with a blue histogram. The fitted function to describe the baseline is also shown with a continuous red line. The periods used to fit the baseline correspond to the blue regions, where the impact of influenza and heat waves is negligible.

The number of deaths for the whole dataset used by the analysis is shown in Figure 1. The periods used for the baseline fit and the fit results are also overlaid. The different excesses due to influenza and heat waves and the COVID waves for the years 2020–2022 are clearly visible.

**Figure 2:**
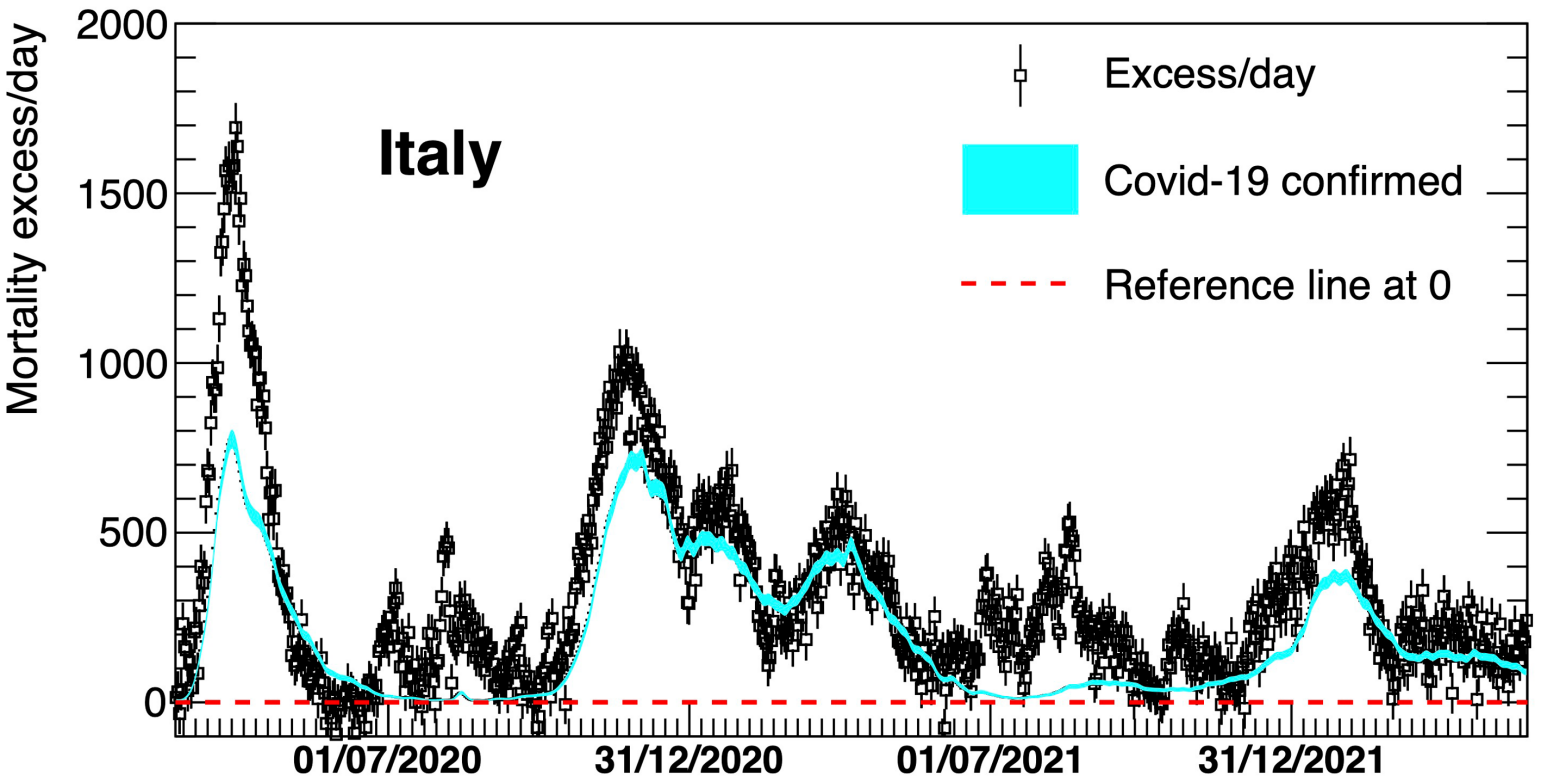
Excess in deaths per day in Italy 23 February 2020–30 April 2022. Results are compared with the official COVID-19 deaths (light blue line). Death excesses are shown with respect to zero, represented with a red dashed line.

In Table 1, the results for the floating parameters in the fits are detailed, then the resulting subtracted distribution is shown in Figure 2. A dashed red line is shown at zero to better visualize the extent of the daily excess of mortality once the background has been subtracted. The excess is compared with the official laboratory-confirmed COVID-19 deaths.

**Table 1:** Results for the floating parameters of the fit to the number of deaths in Italy vs. time shown in Figure 1.

	Fitted values ± 1SE
Offset	1685.9 ± 3.1
Slope	−3.3 ± 1.7
Amplitude	173.3 ± 3.9
Phase	−30.22 ± 0.58

**Figure 3:**
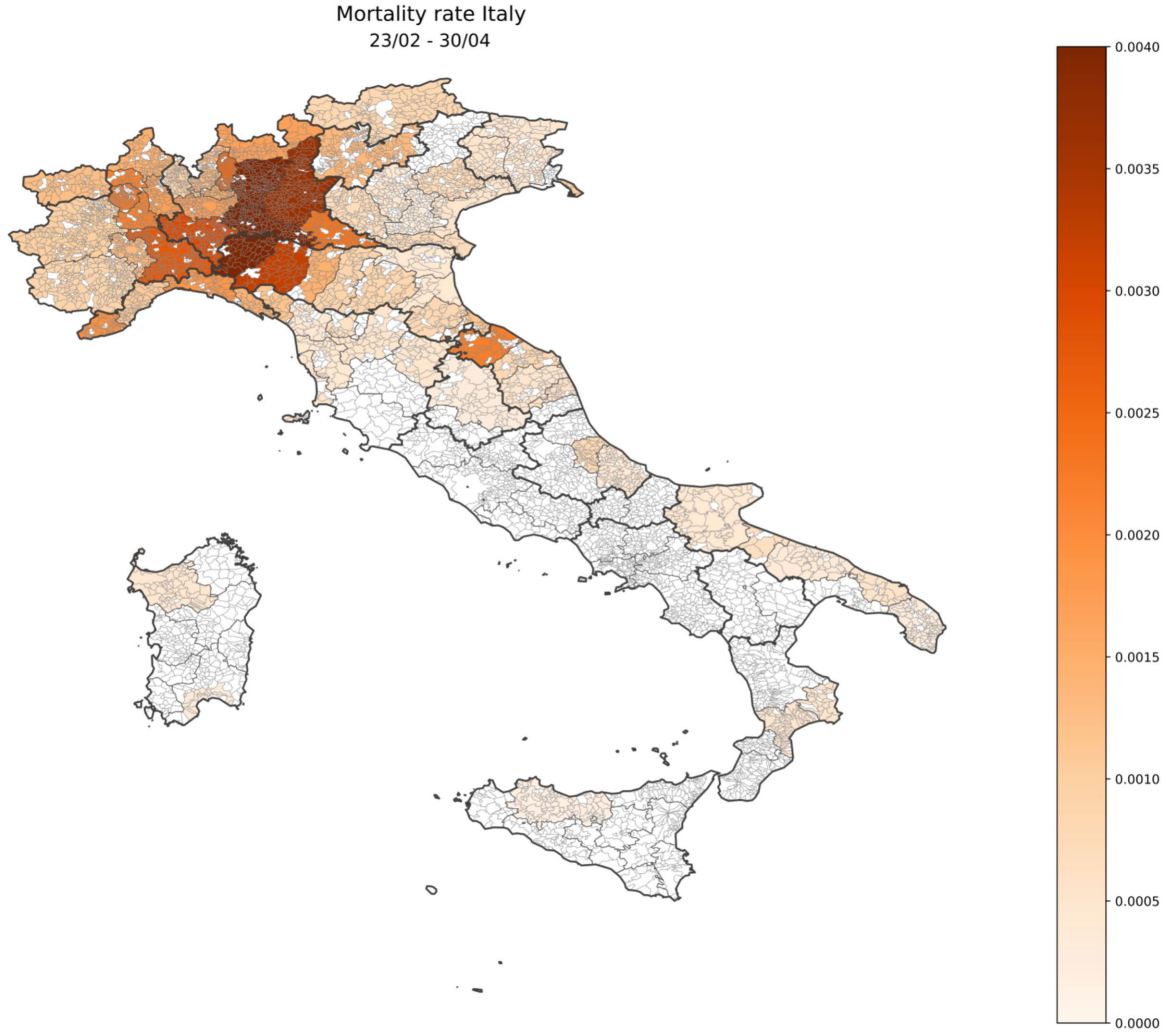
Geographical distribution of Italian mortality excess rate during the first wave of COVID-19 (23 February 2020–30 April 2020) shown in the heat map.

The death rates for Italian provinces obtained by using the same approach for the first wave only of COVID-19 are shown in Figure 3.

**Figure 4:**
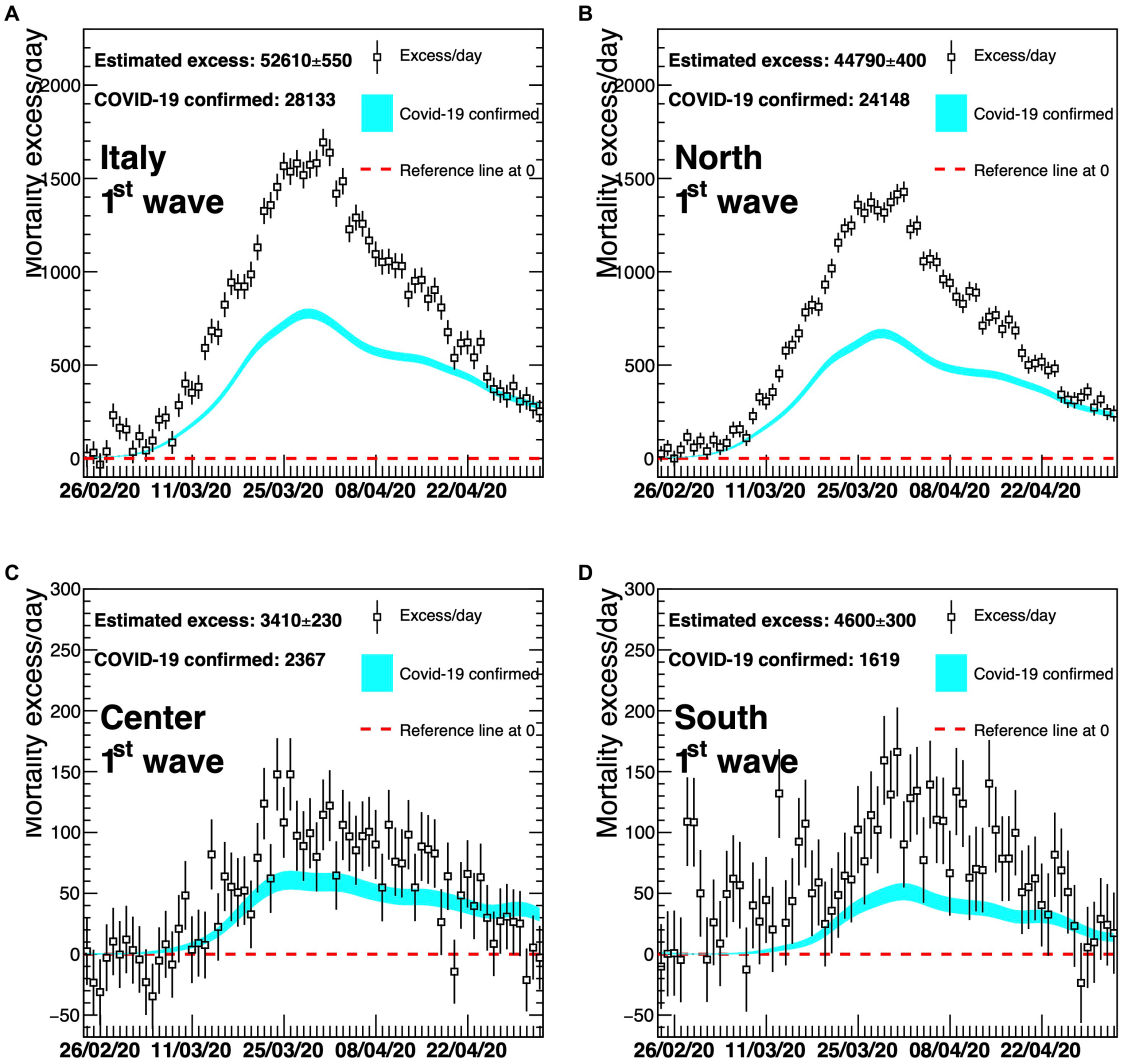
Excess in deaths per day for the first wave of COVID-19 (23 February 2020–30 April 2020) for the whole of Italy **(A)**, Northern Italy **(B)**, Central Italy **(C)**, and Southern Italy **(D)**. Results are compared with the official COVID-19 deaths (light blue line). Death excesses are shown with respect to zero, represented with a red dashed line. The vertical axis defining mortality excess/day uses a scale up to 2300 in the two top graphs **(A,B)** and a scale up to 300 in the two bottom graphs **(C,D)**.

**Figure 5:**
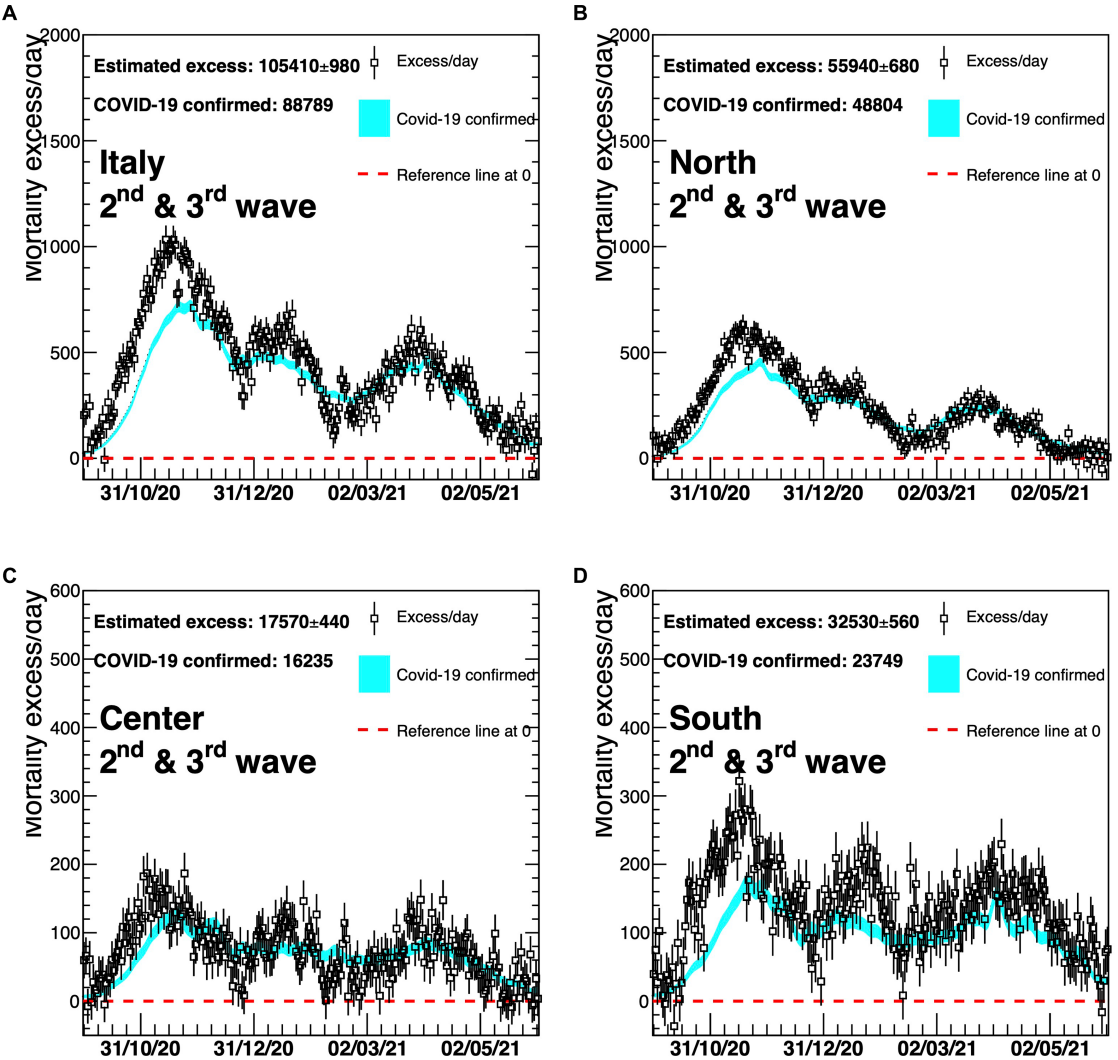
Excess in deaths per day for the second and third waves of COVID-19 (6 October 2020–23 May 2021) for the whole of Italy **(A)**, Northern Italy **(B)**, Central Italy **(C)**, and Southern Italy **(D)**. Results are compared with the official COVID-19 deaths (light blue line). Death excesses are shown with respect to zero, represented with a red dashed line. The vertical axis defining mortality excess/day uses a scale up to 2,000 in the two top graphs **(A,B)** and a scale up to 600 in the two bottom graphs **(C,D)**.

**Figure 6:**
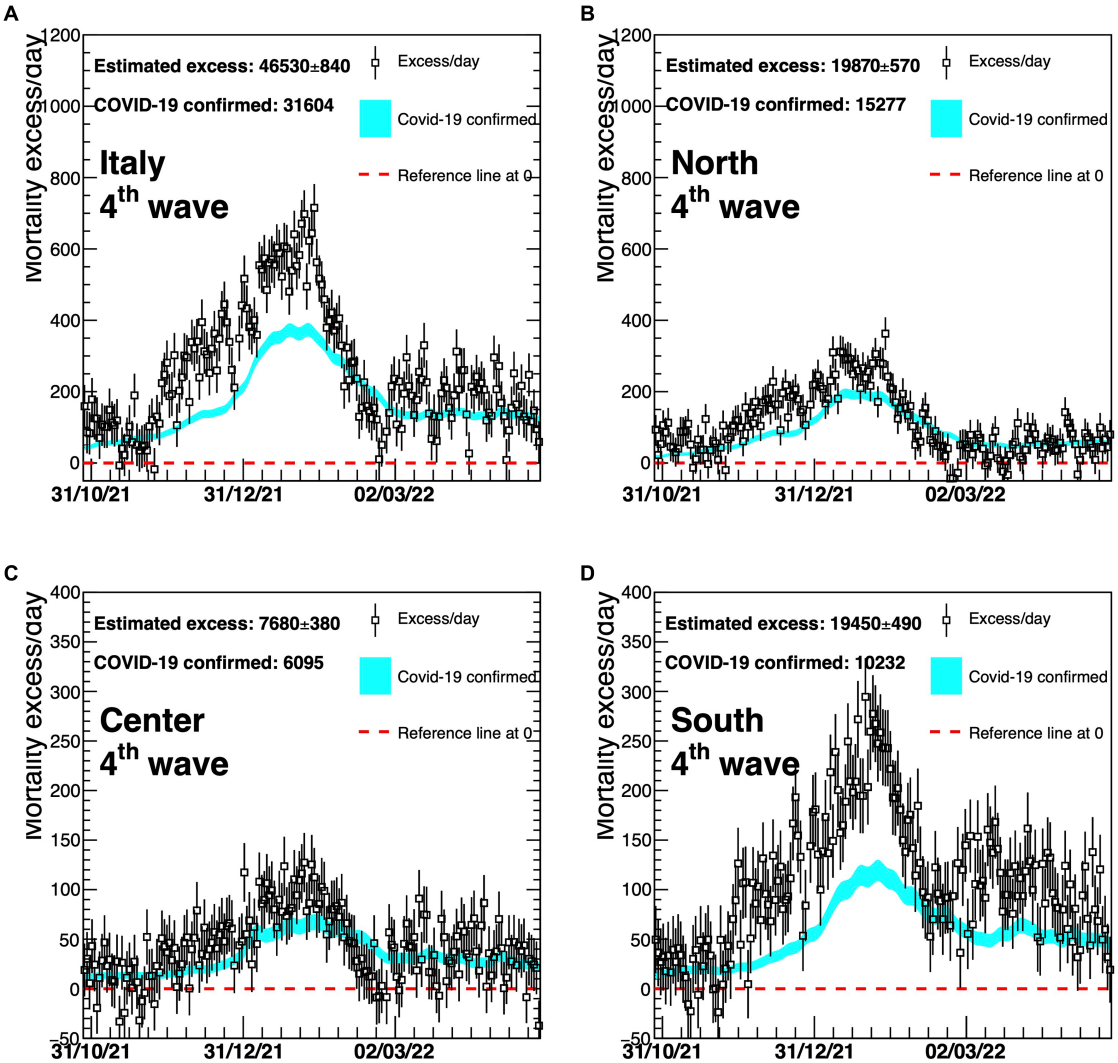
Excess in deaths per day for the fourth wave of COVID-19 (1 November 2021–30 April 2022) for the whole of Italy **(A)**, Northern Italy **(B)**, Central Italy **(C)**, and Southern Italy **(D)**. Results are compared with the official COVID-19 deaths (light blue line). Death excesses are shown with respect to zero, represented with a red dashed line. The vertical axis defining mortality excess/day uses a scale up to 1,200 in the two top graphs **(A,B)** and a scale up to 400 in the two bottom graphs **(C,D)**.

A detailed description by geographical area (North, Centre, and South Islands, as Italy is usually geographically classified) of the four different waves is shown in Figures 4–6. On the graphs, the pattern of the excess mortality is compared with the pattern for the official deaths (blue).

The comparison of the mortality excess with official numbers shows that, during the first wave, there has been an underestimation of COVID-19 deaths, particularly at the beginning of the pandemic and in Northern Italy (Figure 4). The excess of mortality for the first wave started at the end of February 2020, when the first Italian case of COVID-19 was officially reported, and then rapidly increased indicating that, at the beginning of the outbreak, a large fraction of deaths was not identified by the official monitoring system.

Later, the two curves tend to be closer to each other, suggesting that the identification of COVID-19 deaths has improved with time (Figures 5, 6). In particular, Figure 5 reveals a much better agreement between the dotted (excess/day) and the light blue (COVID-19 confirmed) lines although a delay in the time of the official monitoring system is still present at the beginning of the second wave. There is a slightly worse agreement for the Southern part of Italy.

Finally, Figure 6 represents the fourth COVID-19 wave. In this case, Northern and Central Italy show a reasonable agreement, while a large relative underestimation of almost a factor 2 is present for the Southern part of the country, during most time of this wave.

The gap between the curves of the national estimated and officially registered mortality rates shown in panel (A) of Figures 4–6 reflects the geographical deviations described above.

Results in Table 2 indicate that the estimated COVID-19 mortality rate almost doubled the official rate in the first wave for the North (1.60‰ vs. 0.86‰) and nearly tripled it in the South (0.22‰ vs. 0.08‰) even if in the South the magnitude of deaths estimated and occurred was much smaller in absolute values.

**Table 2:** Summary of the death excess results compared with official COVID-19 mortality.

		Estimates of COVID-19 excess of deaths (± uncertainty)*	Official deaths	Estimated COVID-19 mortality rates (per 1,000) (± uncertainty)*	Official mortality rates (per 1,000)	Ratio between estimated and official rates (± uncertainty)*
First wave	North	44,790 ± 400	24,148	1.60 ± 0.02	0.86	1.85 ± 0.02
	Central	3,410 ± 230	2,367	0.28 ± 0.02	0.20	1.44 ± 0.10
	South	4,600 ± 300	1,619	0.22 ± 0.02	0.08	2.84 ± 0.19
	Italy	52,610 ± 550	28,133	0.87 ± 0.01	0.47	1.87 ± 0.02
Second and third waves	North	55,940 ± 680	48,804	2.00 ± 0.02	1.74	1.15 ± 0.01
	Central	17,570 ± 440	16,235	1.47 ± 0.04	1.36	1.08 ± 0.03
	South	32,530 ± 560	23,749	1.62 ± 0.03	1.19	1.37 ± 0.02
	Italy	105,410 ± 980	88,789	1.75 ± 0.02	1.47	1.19 ± 0.01
Fourth wave	North	19,870 ± 570	15,277	0.71 ± 0.02	0.55	1.30 ± 0.04
	Central	7,680 ± 380	6,095	0.64 ± 0.03	0.51	1.26 ± 0.06
	South	19,450 ± 490	10,232	0.97 ± 0.02	0.51	1.90 ± 0.04
	Italy	46,530 ± 840	31,604	0.77 ± 0.01	0.52	1.47 ± 0.03

*The uncertainty related to the estimated excess of deaths and rates is due to the Poissonian error in the counting and the statistical uncertainty of the background subtraction.

During the second and third waves, there was no big difference between estimated and official mortality rates, whereas during the fourth wave not only did the South of Italy display comparatively a very substantial discrepancy (+90%), but was also characterized in this case by a high absolute excess mortality which rose to 42% of the total excess of deaths in Italy from 9% of the first wave.

## 4 Discussion

Accuracy, precision, and reliability are key elements of disease estimates, especially during pandemics when many articles (often without peer review) might lead to the dissemination of flawed studies (25). In dealing with contagious and transmissible diseases, the correct estimate of the extent of deaths is very important for public health professionals and policymakers in order to mitigate the effect of transmission. In the recent COVID-19 pandemic, there was no consensus about which data were more reliable for developing containment measures such as lockdowns, quarantine, isolation, and cordon sanitaire, especially considering the possible negative implications (e.g., on mental health and on children’s education) of such public health policies.

At the beginning of the pandemic, some studies reported crude numbers of COVID-19 deaths as published by National Health Authorities (21), while others expressed doubts about the observed or estimated rates connected to the emergency, the overload of the healthcare system, the avoidance of hospital care because of fear, and the absence of a diagnosis because of dying at home or in nursing homes without a microbiological diagnosis (26, 27).

The results in this study provide a measure of the COVID-19 excess deaths, extending up until 30 April 2022 previous results consistent with ours which were published, using different but comparable excess mortality techniques, on the initial 2020 wave of COVID-19 across Italy or in some specific region (12–15, 18, 19); in particular, our analyses provide novel unbiased estimates of the Italian regional mortality rates across four pandemic waves, based on time series modeling previously adopted also by EuroMOMO (24).

During the first wave, mortality turned out to be much higher than that based on official data, especially at the beginning of the outbreak. In Northern Italy, the excess of mortality was higher than in Central and Southern Italy, but all curves show a very similar shape over time. In each curve, the position of the maximum, a direct consequence of the lockdown is, as expected, very similar, and corresponds to the end of March 2020, that is, approximately 15 days after the enactment of lockdown. Thanks to this containment measure, larger numbers of deaths could be prevented, even though the virus was already circulating and causing deaths in Central and Southern Italy, showing the effectiveness of this difficult policy decision at that moment, to avoid the health system collapse.

Figures 2, 5, 6 show that COVID-19 deaths decreased over time along the second and third waves; a slight rise is observed during the fourth wave coinciding with the Christmas and New Year holiday period, which in Italy are traditionally celebrated with family and friends, circumstances that could have facilitated virus spreading. Moreover, although the time profile of official and estimated mortality data looks almost the same for the second and third waves, there is a delay of roughly 10 days in the observed official data, corresponding to the difference between the maxima of the two distributions. This delay depends on the surveillance system itself which allows some days of delay after death for official communication.

In the fourth wave (Figure 6), there is a difference between the estimated and official deaths as observed in panel (A), probably due to the same gap displayed in panel (D) for Southern Italy. This could be related to different hypotheses: First, the healthcare system may not have coped with the prolonged pressure of the pandemic; second, there could have been an underreporting of official data, once the emergency period was over and vaccinations together with new COVID-19 treatments eased the pandemic fear.

A recommendation for the future could be to implement an Italian computerized statistical death form to avoid the delays connected to the use of paper forms for official death communication to the mortality surveillance system. Moreover, this tool could be more user-friendly than the paper one, helping the physician throughout the data compilation process with specific message boxes.

Finally, our study presents some limitations: There are possible potential biases represented by either a wrong baseline subtraction or by the impact of other causes of death, which can be correlated with the presence of COVID-19; however, the latter does not look dominant, because the baseline-subtracted distributions show the typical exponentially-growing, Gompertz-like shape as for the official ones. In addition, the ratio between mortality excess and official data does not seem to depend on the mortality rate, as it would happen if there was a correlation with the spread of the virus: For instance, in the first wave, this ratio was larger for Southern Italy than for Northern Italy. If such a correlation had been present, we would have observed the opposite effect, as the impact of the virus had been much weaker in Southern Italy.

In future, the spread of transmissible diseases could take advantage of the use of Artificial Intelligence (AI) software support to interpret health data coming from wearables (e.g., breath or cough sounds assessment and saturation analysis) to faster detect individual infections and exploit this information to promptly alert the public health surveillance systems. This shade of digital public health can also be very useful to put into action faster mitigation measures aimed at minimizing the number of cases and deaths, during any future health threats, i.e., not only in the rare case of future relapses of COVID-19, but also for pandemics that may develop in the near future from other viruses, such as Influenza viruses or any new virus that may spillover and that cannot be clearly foreseen at the moment we are writing the article.

In conclusion, the methods we used are to be considered as tools for Public Health professionals to monitor mortality and help detect future emergency scenarios, avoiding the lack of preparedness that characterized the COVID-19 epidemic in Italy.

For Figures 4–6, panel (A) corresponds to upper left, panel (B) to upper right, panel (C) to bottom left, panel (D) to bottom right.

## Data availability statement

Publicly available datasets were analyzed in this study. This data can be found at: https://www.istat.it/it/archivio/240401.

## Author contributions

DR: Conceptualization, Data curation, Formal analysis, Funding acquisition, Methodology, Software, Supervision, Visualization, Writing – original draft. LP: Data curation, Formal analysis, Methodology, Supervision, Writing – original draft, Writing – review & editing. PM: Conceptualization, Data curation, Formal analysis, Methodology, Software, Visualization, Writing – original draft. LS: Formal analysis, Software, Visualization, Writing – original draft. ML: Formal analysis, Supervision, Writing – original draft. MA: Formal analysis, Supervision, Writing – original draft, Writing – review & editing. MC: Formal analysis, Supervision, Writing – original draft, Writing – review & editing.
